# Physiological and Proteomic Analysis of *Penicillium digitatum* in Response to X33 Antifungal Extract Treatment

**DOI:** 10.3389/fmicb.2020.584331

**Published:** 2020-11-06

**Authors:** Shu-Hua Lin, Pan Luo, En Yuan, Xiangdong Zhu, Bin Zhang, Xiaoyu Wu

**Affiliations:** ^1^College of Bioscience and Bioengineering, Jiangxi Agricultural University, Nanchang, China; ^2^Jiangxi Engineering Laboratory for the Development and Utilization of Agricultural Microbial Resources, Nanchang, China; ^3^Collaborative Innovation Center of Postharvest Key Technology and Quality Safety of Fruits and Vegetables in Jiangxi Province, Nanchang, China; ^4^College of Pharmacy, Jiangxi University of Traditional Chinese Medicine, Nanchang, China

**Keywords:** proteomic, antifungal mechanism, *Penicillium digitatum*, X33 antifungal metabolite, citrus green mold

## Abstract

*Penicillium digitatum* is a widespread pathogen among Rutaceae species that causes severe fruit decay symptoms on infected citrus fruit (known as citrus green mold). The employment of fungicides can effectively control the citrus green mold, significantly reducing agricultural economic loss. In this study, we found that the X33 antifungal extract produced by *Streptomyces lavendulae* strain X33 inhibited the hyphae polarization of *P. digitatum*. Additionally, physiological and proteomic analysis strategies were applied to explore the inhibitory mechanism of the X33 antifungal extract of the *S. lavendulae* strain X33 on the mycelial growth of *P. digitatum*. A total of 277 differentially expressed proteins, consisting of 207 upregulated and 70 downregulated, were identified from the comparative proteomics analysis. The results indicated that the X33 antifungal extract induced mitochondrial membrane dysfunction and cellular integrity impairment, which can affect energy metabolism, oxidative stress, and transmembrane transport. The improved alkaline phosphatase activity and extracellular conductivity, increased H_2_O_2_ and malondialdehyde contents, and inhibition of energy, amino acid, and sugar metabolism indicated that the oxidative stress of *P. digitatum* is induced by the X33 antifungal extract. These findings provided insight into the antifungal mechanism of the X33 antifungal extract against *P. digitatum* by suggesting that it may be an effective fungicide for controlling citrus postharvest green mold.

## Introduction

Citrus fruits are vulnerable to pathogen like *Penicillium digitatum*, *Penicillium italicum*, and *Geotrichum citri-aurantii* during the postharvest storage phase (Moraes Bazioli et al., 2019). In particularly, *P. digitatum* serves as a universal postharvest pathogenic fungi, caused approximately 90% enormous economic loss in the field of agriculture (Zhu et al., 2017). Currently, developing efficient approaches to suppress the postharvest diseases of citrus has become an active field of research, in which chemical methods occupy a large proportion (Wuryatmo et al., 2014). The method is effective in controlling the postharvest disease, but it also pose sustained threats to food safety, human health, and the ecology of environments. To overcome these challenges, relatively safe and efficient biocontrol strategies using microorganisms and their metabolites have been extensively explored for citrus fruit’s postharvest diseases. *Bacillus* spp., *Streptomyces* spp., *Pseudomonas* spp., and *Trichoderma* spp. are currently regarded as effective antagonistic microbes for inhibiting the growth of *P. digitatum* in citrus fruit (Wu et al., 2019).

*Streptomyces* spp. are important biocontrol microorganisms that play a crucial part in the prevention and control of postharvest fruit diseases owing to their superiority in biobased production of numerous chemicals, which exerts diverse structures and impressive pharmaceutical activity (Chen et al., 2018). For example, *Streptomyces* M-Z18 (Chen et al., 2015), *Streptomyces albulus* NK660 (Geng et al., 2014), and *Streptomyces ahygroscopicus* STZ (Chen et al., 2019c) produce the globally used food preservative ε-poly-L-lysine. In a previous study, we isolated, identified, and named the *Streptomyces lavendulae* strain X33 (CCTCC M2013163), which exhibited powerful biocontrol effects on *P. digitatum* by secreting an antifungal substance similar to ε-polylysine (Wu et al., 2014). However, the molecular mechanism of how the active substance affects the cell growth of *P. digitatum* requires to be uncovered.

In practice, protein expression changes in response to the X33 antifungal extract of pathogens are crucial for explaining the inhibition mechanism of the X33 antifungal extract. Recently, numerous techniques with aim to explore the physiological mechanism and identifying the expression of related proteins in response to environment stress, including two-dimensional polyacrylamide gel electrophoresis (Bittarello et al., 2019), isobaric tags for relative and absolute quantitation (iTRAQ) (Fontarigo et al., 2020), and label-free quantitative proteomics (Yu et al., 2020), have been developed. Of these, iTRAQ based protein quantitative analysis approach serves as the most useful tools to identify protein expression changes in microbes under various conditions (Liu et al., 2017). For example, iTRAQ can easily find out the quantity of proteins and allows quantitative analysis of multiple samples from diverse sources simultaneously. However, rarely studies have considered the *P. digitatum* proteome in response to active substances. Therefore, this study aimed to identify differentially expressed proteins (DEPs) in *P. digitatum* stimulated by active substances via iTRAQ approach and explore the intrinsic molecular mechanism of the X33 antifungal extract on *P. digitatum*.

## Materials and Methods

### Antifungal Activity Evaluation of X33 Antifungal Extract

X33 antifungal extract is a mixture (the main component is ε-three poly-L-lysine) that isolated from fermentation supernatant of *S. lavendulae* strain X33 (stored in the typical culture preservation center of China, No. CCTCC M2013163) (Wu et al., 2014). The activity of the X33 antifungal extract against *P. digitatum* was tested using the Oxford cup method, as describe previously (Khawaja et al., 2018). *P. digitatum* was cultured in potato dextrose agar (PDA) medium (harboring leaching solution of potato 200 g, glucose 20 g, agar 20 g, per liter water) at 28°C. Spores was diluted to achieve a concentration of approximately 10^7^ spores/mL with a blood cell counter. Under sterile conditions, 1 mL of spore suspension was transformed into 50 mL of PDA medium and poured into a plate for condensation, and an Oxford cup was placed in the center of the medium. Then, 200 μL (0, 1.2, 2.4, and 4.8 mg/mL) of each X33 antifungal extract was added to each cup, which were subsequently cultivated at 28°C for 48 h. After incubation, the transparent zone across the Oxford cup was caused by the antifungal effect of the X33 metabolite. The diameter of the zone of inhibition (ZOI; mm) was determined using calipers in triplicate and stated as mean ± standard error.

### iTRAQ Analysis

Fresh spores were cultured under shaking in potato dextrose broth (PDB) for 3 days at 28°C and 160 rpm. Diverse content (0 and 1.2 mg/mL) of the X33 antifungal extracts were devoted into the fermentation cultures at 48 h. After vacuum filtration, the hyphae were washed with deionized water and collected. The harvested mycelia were quickly frozen in liquid nitrogen for iTRAQ analysis. Samples preparation was conducted twice independently with three biological repeats as described previously (Zhou T. et al., 2020). Protein preparation, digestion, isotope labeling, LC-MS/MS analysis, and identification were carried out in accordance with the standard procedure described previously (Ma et al., 2020; Zhou T. et al., 2020). The DEPs were identified using ratios with *p* < 0.05 and fold-changes of >1.2 or <0.83. The protein functional annotation and classification was performed by using the Blast2GO program and Kyoto Encyclopedia of Genes and Genomes (KEGG) database^1^.

### Test of Plasma Membrane Integrity

The extracellular conductivity of *P. digitatum* was estimated according to previous study (Tao et al., 2019) with modulations on employing an electrical conductivity meter (model ST3100c/F, Ohaus Co., New Jersey, United States) (Chen et al., 2019d). Two grams of the fresh mycelium collected at 48 h was re-suspended in 50 mL PDB, which X33 antifungal extract was poured and adjusted to 0, 1.2, 2.4, and 4.8 mg/mL. The extracellular electric conductivity was measured at 0, 1, 2, 3, 4, and 5 h, and expressed as the amount of extracellular conductivity (μs/cm) (Chen et al., 2019d).

### Determination of Alkaline Phosphatase (AKP) Activity and Ergosterol Content

The AKP activities of *P. digitatum* mycelia with diverse content of X33 antifungal extract treatments were processed by using a UV-2450 UV/Vis spectrophotometer [SHIMADZU international trade (Shanghai) Co., Ltd., Shanghai, China] and an AKP kit (Jiancheng Bioengineering Research Institute Co., Ltd., Nanjing, China) according to the manufacturer’s instructions (OuYang et al., 2019).

*Penicillium digitatum* was cultured in a 250 mL Triangular flask containing 40 mL PDB broth at 28°C and 160 rpm. At 48 h of cultivation process, various concentrations (0, 1.2, 2.4, and 4.8 mg/mL) of the X33 antifungal extract were added to the culture mediums. After centrifuging at 12,000 rpm for 10 min, the mycelium was washed with sterile water and dried on filter paper for measuring the ergosterol contents. The determination of ergosterol was measured using the way described previously (Silva et al., 2010).

### Effect of the X33 Antifungal Extract on *P. digitatum* Morphology

After treatment with the X33 antifungal extract of the *S. lavendulae* strain X33, the samples of *P. digitatum* micromorphology were visualized by scanning electron microscopy (SEM; FEI Quanta FEG 250, United States), according to the method described previously (Ju et al., 2020).

### Measurement of Oxidative Stress Parameters in *P. digitatum* Cells

After induced by X33 antifungal extract, the cultivated samples were collected at logarithmic phase and subsequently applied to determine the hydrogen peroxide (H_2_O_2_) and malondialdehyde (MDA) contents. The MDA content was estimated by using thiobarbituric acid approach as described previously (Zhiheng et al., 2014). The extraction and evaluation of H_2_O_2_ in *P. digitatum* hyphae were conducted according to the method reported previously (Chen et al., 2019d). Frozen mycelia samples were homogenized in 5 mL of ice-cold acetone and centrifuged at 15,000 × *g* for 20 min. The supernatant was collected for subsequent analysis. Determination of H_2_O_2_ content was performed using a H_2_O_2_ detection kit (Nanjing Jiancheng Bioengineering Institute, Nanjing, China), according to the manufacturer’s instructions (Sally et al., 2017).

### Pyruvate Measurements

The pyruvate content was measured as per the method of Ho et al. (2020), with slight modifications. Hyphae were homogenized in ice-cold PBS (10 mM, pH 7.0) by quartz sand. Then, the homogenates were centrifuged at 15, 000 × *g* for 20 min at 4°C. The supernatants were then collected for pyruvic acid determination.

## Results

### The Effect of the X33 Antifungal Extract on the Cell Growth of *P. digitatum*

**FIGURE 1:**
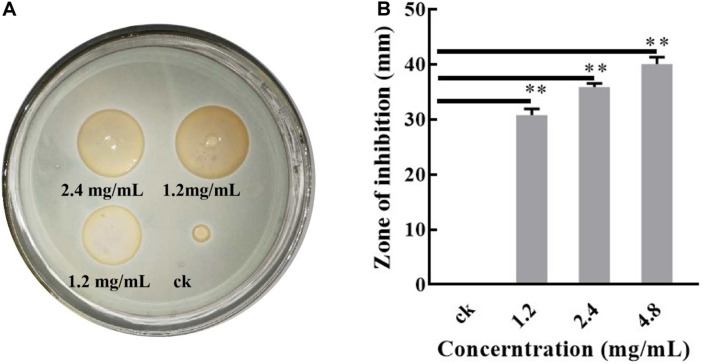
Antibacterial test of the X33 antifungal extract against *P. digitatum*. Results of standard deviations present in three individual experiments. **p < 0.01.

Previously, a novel citrus green mold biocontrol strain, *S. lavendulae* X33, was isolated from its antagonism evaluation for *P. digitatum*. After long-term studies, we found that the biological control characteristics of this strain were probably related to the secretion of an active substance, which is identified as ε-three poly-L-lysine. The deduced process is listed in the supporting materials. Interestingly, this X33 antifungal extract exhibits superior activity to ε-poly-L-lysine produced by *Streptomyces albus*. To further investigate its mechanism, antimicrobial effects of the X33 antifungal extract were evaluated on the mycelial radial growth of *P. digitatum*. Results from the Oxford cups assay indicated that the ZOIs formed by the X33 antifungal extract (1.2, 2.4, and 4.8 mg/mL) on *P. digitatum* were in the ranges of 30.82 ± 0.90, 35.94 ± 0.53, and 40.01 ± 1.02 mm, respectively (Figure 1). The fungistatic activity of the X33 antifungal extract on *P. digitatum* is dose-dependent; the higher the concentration of the X33 antifungal extract, the higher the antifungal activity against *P. digitatum*.

### Comparative Proteomics Analysis of *P. digitatum* Treated With the X33 Antifungal Extract

**FIGURE 2:**
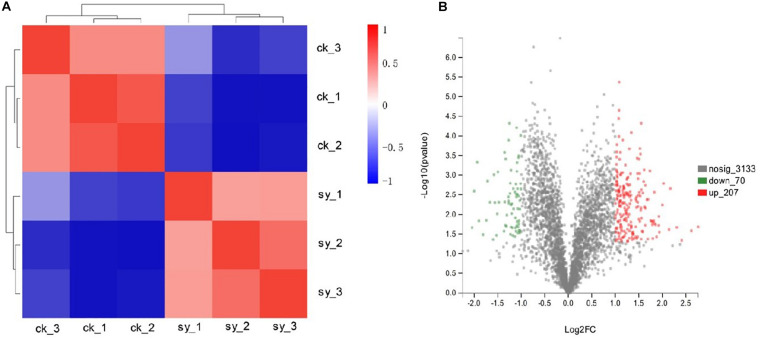
Proteins in *P. digitatum* cells under 1.2 mg/mL X33 antifungal extract treatment. **(A)** Expression changes of major DEPs of *P. digitatum* cells. **(B)** Cluster analysis of differentially expressed proteins.

Through fungistatic activity analysis, we found that the X33 antifungal extract can inhibit the mycelial radial growth of *P. digitatum*. However, the intrinsic mechanism of how the X33 antifungal extract induces the cell death of *P. digitatum* remains to be determined. Uncovering this mechanism can not only promote the application of the X33 antifungal extract, but also provide an important reference for the development of new food preservatives. Therefore, proteomic analysis was processed to depict a complete picture of the protein’s expression of *P. digitatum* affected by the X33 antifungal extract, which is commonly used for the exploration of the antimicrobial mechanism of active compounds. 3410 proteins were identified by iTRAQ analysis with the aim of detecting DEPs between control and treatment groups (Supplementary Table S1). Among them, 70 downregulated and 207 upregulated proteins were found to be significantly different between the X33 antifungal extract-treated groups and control groups, according to the screening criteria (Table 1, Figure 2, and Supplementary Table S2). Clustering analysis was performed to compare the expression patterns of DEPs in six groups (Figure 2B). The expression patterns of ck-1 and sy-1, ck-2 and sy-2, and ck-3 and sy-3 were gathered into clusters, which reflected a remarkable difference in the protein expression between the control group and X33 antifungal extract-treated samples.

**TABLE 1:** Identification of differentially expressed proteins in *P. digitatum* after X33 antifungal extract treatment.

**Uniprot no.**	**NCBI accession**	**Protein name**	**Fold change**	***P*-value**	**Regulated type**	**Protein MW [kDa]**
TRINITY_DN433_c0_g3_i2_orf1	gi| 557729604	Ecdysteroid kinase; phosphotransferase enzyme family	2.76	0.02	Up	47.4
TRINITY_DN4251_c0_g1_i5_orf1	gi| 145334271	Histone H3.3	2.62	0.03	Up	15.4
TRINITY_DN3068_c0_g1_i1_orf1	gi| 700481483	Translation initiation factor 1A	2.31	0.02	Up	17.1
TRINITY_DN4415_c0_g1_i1_orf1	gi| 255948382	Pc22g09460, partial	2.41	0.05	Up	18
TRINITY_DN9730_c0_g1_i1_orf1	gi| 952553567	40S ribosomal protein S9	2.17	0.00	Up	23.5
TRINITY_DN4101_c0_g1_i1_orf1	gi| 901823486	Phosphopyruvate hydratase	2.14	0.02	Up	8.8
TRINITY_DN4163_c0_g1_i2_orf1	gi| 119482768	Phosphatidylglycerol specific phospholipase C,	1.92	0.02	Up	50.5
TRINITY_DN8868_c0_g1_i1_orf1	gi| 145247997	ATP phosphoribosyltransferase	1.54	0.00	Up	6.54
TRINITY_DN3396_c0_g1_i1_orf1	gi| 121713168	Vacuolar ATPase proteolipid subunit c	1.79	0.02	Up	15.9
TRINITY_DN1388_c0_g2_i1_orf1	gi| 859268431	Putative C-4 methylsterol oxidase	1.56	0.01	Up	34.1
TRINITY_DN6766_c0_g1_i1_orf1	gi| 584410839	Catalase B	1.55	0.01	Up	80.4
TRINITY_DN10398_c0_g1_i1_orf1	gi| 351724251	Translationally controlled tumor protein homolog	2.00	0.01	Up	4.73
TRINITY_DN9371_c0_g1_i1_orf1	gi| 731318940	Phosphoglycerate kinase	1.94	0.03	Up	42.8
TRINITY_DN1464_c0_g1_i1_orf1	gi| 859262625	Endoglucanase	1.64	0.02	Up	52.6
TRINITY_DN5267_c0_g2_i1_orf1	gi| 983780712	Glucose oxidase	1.52	0.02	Up	4.41
TRINITY_DN9235_c0_g1_i1_orf1	gi| 915165989	Phospholipase C	1.46	0.00	Up	68.7
TRINITY_DN784_c0_g1_i7_orf1	gi| 859267626	External NADH-ubiquinone oxidoreductase	1.47	0.00	Up	64.8
TRINITY_DN2583_c0_g1_i2_orf1	gi| 859270273	ATP synthase subunit H	1.50	0.01	Up	9.76
TRINITY_DN14971_c0_g1_i1_orf1	gi| 859269367	Aldose 1-epimerase	1.20	0.04	Up	49.1
TRINITY_DN11949_c0_g1_i1_orf1	gi| 859269359	Glucanosyltransferase	1.43	0.02	Up	47.7
TRINITY_DN255_c0_g1_i1_orf1	gi| 859266705	Cell wall glucanase	1.12	0.04	Up	48.1
TRINITY_DN2262_c0_g1_i2_orf1	gi| 358373893	NADH-ubiquinone oxidoreductase 12 kda subunit	2.42	1.27	Up	11.6
TRINITY_DN5697_c0_g1_i1_orf1	gi| 584411575	Chitin-binding, type 1	2.71	1.44	Up	33.5
TRINITY_DN15423_c0_g1_i1_orf1	gi| 859261761	Single-stranded DNA-binding protein	2.60	1.38	Up	9.63
TRINITY_DN3834_c0_g1_i2_orf1	gi| 995934404	Acyl carrier protein	1.45	0.00	Up	15.2
TRINITY_DN2324_c0_g1_i4_orf1	gi| 859262943	1,3-beta-glucanosyltransferase	1.30	0.04	Up	56.4
TRINITY_DN13166_c0_g1_i1_orf1	gi| 859271438	Cytochrome P450	1.24	0.01	Up	11
TRINITY_DN2837_c0_g1_i5_orf1	gi| 952549797	NADH-ubiquinone oxidoreductase 20.9 kDa subunit	1.10	0.00	Up	20.6
TRINITY_DN4998_c0_g1_i1_orf1	gi| 859266290	Chitin synthase V	1.15	0.01	Up	206.3
TRINITY_DN5267_c0_g1_i1_orf1	gi| 375244000	Glucose oxidase	1.40	0.04	Up	10.3
TRINITY_DN13882_c0_g1_i1_orf1	gi| 350638428	CDP-alcohol phosphatidyltransferase	1.23	0.00	Up	26.6
TRINITY_DN3973_c0_g1_i1_orf1	gi| 859270216	Beta-glucan synthesis-associated protein	1.02	0.00	Up	73.4
TRINITY_DN1690_c0_g1_i2_orf1	gi| 859259834	Serine/threonine-protein kinase RIO1	1.24	0.01	Up	64.3
TRINITY_DN15356_c0_g1_i1_orf1	gi| 859264692	Acetyl-coenzyme A transporter 1	1.08	0.00	Up	64.1
TRINITY_DN2077_c0_g1_i1_orf1	gi| 525588153	NADH dehydrogenase	1.04	0.00	Up	8.4
TRINITY_DN1731_c0_g1_i1_orf1	gi| 859260053	Beta-glucosidase	1.07	0.00	Up	46.5
TRINITY_DN212_c0_g1_i2_orf1	gi| 859268618	Pyruvate carboxylase	−1.01	0.00	Down	131.1
TRINITY_DN15435_c0_g1_i1_orf1	gi| 859263266	Acyl-CoA dehydrogenase	−1.07	0.02	Down	57.4
TRINITY_DN15038_c0_g1_i1_orf1	gi| 145258080	acetyl-CoA C-acyltransferase	−1.22	0.02	Down	12.4
TRINITY_DN1583_c0_g1_i1_orf1	gi| 859271617	Alcohol dehydrogenase	−1.12	0.02	Down	36.5
TRINITY_DN4764_c0_g1_i1_orf1	gi| 859265321	Succinate dehydrogenase	−1.34	0.02	Down	67.1
TRINITY_DN13734_c0_g1_i1_orf1	gi| 145248778	Peroxisomal hydratase-dehydrogenase-epimerase	−1.32	0.02	Down	28.2
TRINITY_DN11802_c0_g1_i1_orf1	gi| 145258080	Acetyl-CoA C-acyltransferase	−1.51	0.01	Down	27.4
TRINITY_DN5777_c0_g1_i1_orf1	gi| 859260572	3-beta hydroxysteroid dehydrogenase/isomerase family protein	−1.72	0.00	Down	37.9
TRINITY_DN12283_c0_g1_i1_orf1	gi| 859264108	Peroxidase	−1.60	0.04	Down	83
TRINITY_DN133_c0_g2_i1_orf1	gi| 145237624	Superoxide dismutase	−1.52	0.03	Down	16
TRINITY_DN5714_c0_g1_i1_orf1	gi| 859260252	D-isomer specific 2-hydroxyacid dehydrogenase, NAD binding domain	−2.04	0.02	Down	9.25

### Functional Annotation of DEPs

To explore the functionality and biological processes relate to the identified DEPs in the control sample and X33 antifungal extract-treated sample, gene ontology (GO) analysis and KEGG enrichments were processed that systematic evaluated the biological functions and meaning of these proteins in life activities. As a result, 235 proteins were sub-categorized into 95 hierarchically structured GO classifications. Additionally, 152 proteins identified for differential metabolic pathways by KEGG were sub-categorized into 61 classifications.

#### GO Annotation of DEPs

**FIGURE 3:**
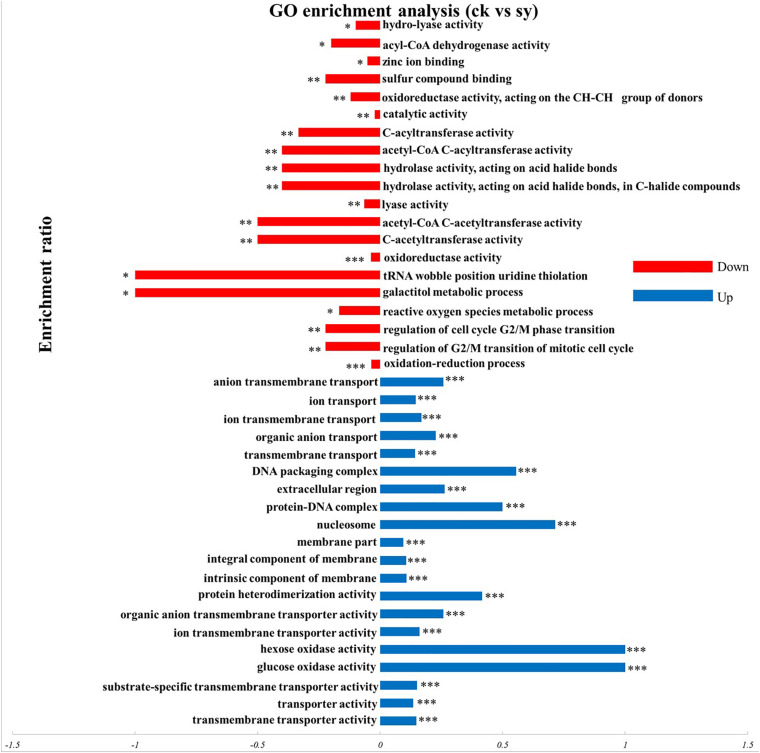
GO enrichment analysis of DEPs in *P. digitatum* cells. *p < 0.05, **p < 0.01, ***p < 0.001.

According to the GO enrichment analyses of DEPs, majority of upregulated DEPs were contributed to transmembrane transporter activities participated in the molecular function category. Categorization of the identified proteins based on cellular components implied that the most abundant proteins were part of the intrinsic components of membranes, integral components of membranes, and membrane parts. A certain amount of proteins was belonging to the biological process. The most abundant subclasses of proteins were transmembrane, organic anion, and ion transmembrane transporters (Figure 3). For the downregulated DEPs, oxidoreductase activity, C-acetyltransferase activity, and acetyl-CoA C-acetyltransferase activity, etc. were the most abundant associated with the molecular function. Furthermore, numerous enrichment terms in the biological process were associated with the oxidation-reduction process, regulation of G2/M transition of the mitotic cell cycle, regulation of the cell cycle G2/M phase transition. Consequently, it can be acknowledged that multitude of proteins in *P. digitatum* induced by X33 antifungal extract treatment may be involved in diverse metabolic and cellular processes.

#### Pathway Annotation

**FIGURE 4:**
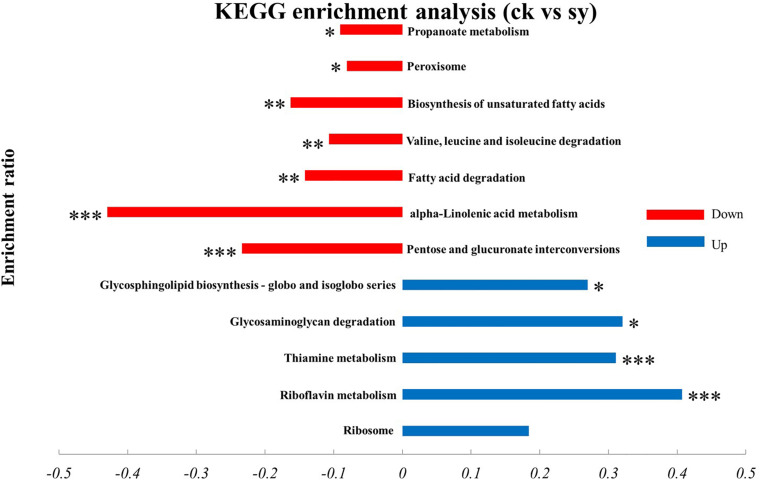
KEGG enrichment analysis of DEPs in *P. digitatum* cells. *p < 0.05, **p < 0.01, ***p < 0.001.

Result from KEGG pathway enrichment analysis indicated that upregulated proteins were impressively enriched in five pathways (*p* < 0.05; Figure 4), of which 16 proteins were enriched in the “Ribosome (map03010)” pathway. Meanwhile, five proteins were enriched in the “Thiamine metabolism (map00730)” pathway and four in the “Riboflavin metabolism (map00740)” pathway. However, only two proteins were enriched in the “Glycosphingolipid biosynthesis globo and isoglobo series (map00603)” and “glycosaminoglycan degradation (map00531)” pathways, respectively. Downregulated genes were significantly enriched in seven pathways (*p* < 0.05), most of which were involved in the “pentose and glucuronate interconversions (map00040)” pathway. Pathways related to “alpha-Linolenic acid metabolism (map00592),” “fatty acid degradation (map00071),” “biosynthesis of unsaturated fatty acids (map01040),” “Peroxisome (map04146),” “propanoate metabolism (map00640),” and “valine, leucine and isoleucine degradation (map00280)” were also significantly enriched.

### Effects of the X33 Antifungal Extract on the Cell Integrity in *P. digitatum*

#### Effects of the X33 Antifungal Extract on the Extracellular Conductivity in *P. digitatum*

**FIGURE 5:**
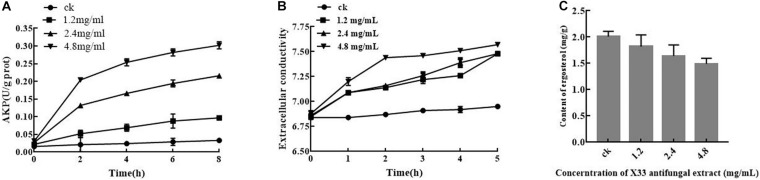
Effects of X33 antifungal extract incubation on cell integrity of *P. digitatum*. **(A)** Extracellular conductivity. **(B)** Alkaline phosphatase activity of *P. digitatum*. **(C)** Ergosterol contents.

Results from quantitative proteomics analysis indicated that the X33 antifungal extract inhibited the cell growth of *P. digitatum*, which is probably related to the destruction of cell membrane integrity, which plays a crucial role in cell activities. Therefore, measuring extracellular conductivity was undertaken to determinate the damage to membrane permeability (Chen et al., 2019b). As illustrated in Figure 5A, the extracellular conductivities of the *P. digitatum* cells were 7.48 ± 0.01, 7.4 ± 0.02, and 7.52 ± 0.03 μs/cm for 5 h after incubation with the X33 antifungal extract at concentrations of 1.2, 2.4, and 4.8 mg/mL, respectively. These values are remarkable higher than that obtained in the control group (6.95 ± 0.01 μs/cm). A stable level of extracellular conductivity was observed in the control, while the conductivity impressive generally improved with the prolonged treatment duration. Meanwhile, a significant difference in the extracellular conductivity was observed among the treatment groups. It can be concluded that the addition of the X33 antifungal extract improved the extracellular conductivity of *P. digitatum* cells.

#### The Effects of the X33 Antifungal Extract on the AKP Activities in *P. digitatum*

The cell wall is a flexible structure that offers protection to the cell and is, thus, essential for filamentous fungal permeability. AKP is an enzyme produced in the cytoplasm that permeates into the periplasmic space (Yang et al., 2016). Generally, AKP is released from fungal cells with impaired cell wall permeability. The AKP activity of the control group was not changed during the whole incubation phase. In contrast, the AKP activity of *P. digitatum* suspensions with treatment (1.2, 2.4, and 4.8 mg/mL) of the X33 antifungal extract rapidly increased in the first hour of exposure, followed by a moderate ascending trend in the subsequent stage (Figure 5B). After 8 h of exposure, the AKP activities in treatment groups with 1.2, 2.4, and 4.8 mg/mL of the X33 antifungal extract were 0.097, 0.216, and 0.302 U/g prot, respectively, which were significantly higher (*p* < 0.05) than in the control (0.033 U/g prot). The increased AKP activity represents increased damage to the cell walls of *P. digitatum*.

#### Effect of X33 Antifungal Extract on Ergosterol Formation in *P. digitatum*

As an essential sterol ingredient of the filamentous fungi cell membranes, ergosterol plays a crucial part in maintaining cell function and integrity. To investigate the influence of *S. lavendulae* strain X33 on ergosterol synthesis, the *P. digitatum* mycelium was treated with several concentrations (0, 1.2, 2.4, and 4.8 mg/mL, respectively) of the antifungal metabolite from *S. lavendulae* strain X33. By comparison with the control group, it can be acknowledged that improved concentration of the X33 antifungal extract would cause a significantly decline of ergosterol formation in *P. digitatum* mycelium (Figure 5C).

### Morphology Changes of *P. digitatum* Under X33 Antifungal Extract Treatment

**FIGURE 6:**
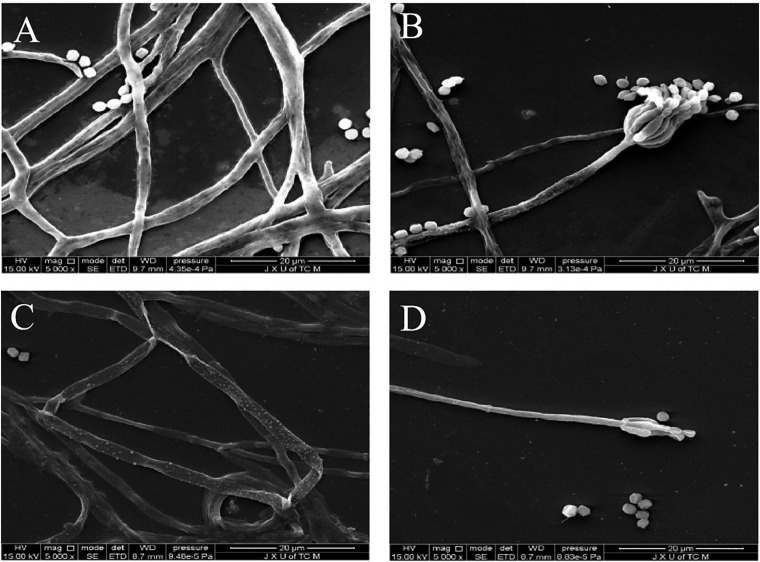
Scanning electron microphotographs of *P. digitatum*. **(A)** Untreated mycelia. **(B)** Untreated spores. **(C)** Mycelia treated with the X33 antifungal extract at 1.2 mg/mL. **(D)** Spores treated with the X33 antifungal extract at 1.2 mg/mL.

Results of SEM images indicate *P. digitatum* without X33 antifungal extract treatment exhibited typical morphology including regular, homogenous, and robust hyphae of a constant diameter, a smooth surface, and contained full cytoplasm (Figure 6A). The conidiophores had normal, plump, and homogenous morphology (Figure 6B). When *P. digitatum* was subjected to the X33 antifungal extract, the hyphae became seriously exfoliated, shriveled, with a rupture and wrinkled surface, distorted, cytoplasm agglutination, and even extravasation (Figure 6C). The shape of the conidial peduncle was abnormal, and the spore yield decreased (Figure 6D).

### The Effects of the X33 Antifungal Extract on Lipid Peroxidation in *P. digitatum*

**FIGURE 7:**
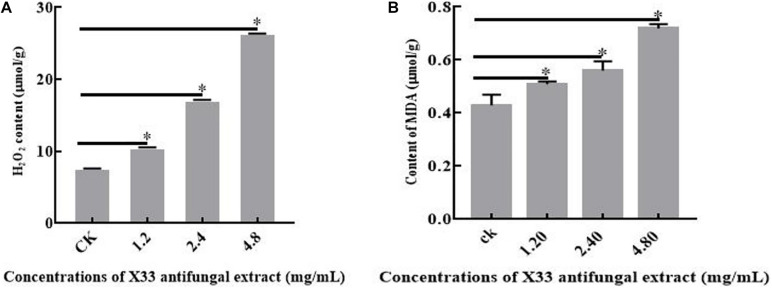
Effects of X33 antifungal extract incubation on the oxidative stress of mycelia of *P. digitatum*. **(A)** Lipid Peroxidation. **(B)** H_2_O_2_ content. *p < 0.05.

Lipid peroxidation can obviously affect cell membrane structure and function, causing biomacromolecules to form crosslinking polymerization, and subsequently induced the cell death (Yang et al., 2019). MDA is applied to evaluate the levels of lipid peroxidation and cell membrane destruction as a biochemical index (Zhang et al., 2020). Therefore, to further investigate cell membrane damage, the lipid peroxidation levels of *P. digitatum* with the treatment of the X33 antifungal extract were compared by measuring MDA contents. A positive correlation was observed between MDA production and the X33 antifungal extract concentration (*p* < 0.05, Figure 7A). When the incubation concentration of the X33 antifungal extract was increased from 1.2 to 2.4 mg/mL, the MDA contents of *P. digitatum* increased from 0.51 to 0.56 μmol/g. Meanwhile, the MDA contents of *P. digitatum* incubated with 4.8 mg/mL of X33 antifungal extract reached 0.72 μmol/g, which was 1.67-fold higher than that of the control group. These results suggested that the X33 antifungal extract induced the lipid peroxidation and cell membrane damage of *P. digitatum*.

### The Effects of X33 Antifungal Extract Treatment on H_2_O_2_ Content

It can be widely acknowledged that the equilibrium between reactive oxygen species (ROS) production and antioxidant defense decides the level of oxidative stress (Tao et al., 2019). H_2_O_2_ is frequently identified as one of the major metabolites of ROS owing to its ability in the production of more reactive species, bring about oxidative stress, and reduce the viability of cells. The oxidative stress levels of *P. digitatum* with X33 antifungal extract treatment were determined by measuring H_2_O_2_ contents. With the increased content of the X33 antifungal extract, the H_2_O_2_ concentrations in *P. digitatum* presented a significant increasing trend (Figure 7B). When the *P. digitatum* hyphae were exposed to 4.8 mg/mL of the X33 antifungal extract, the H_2_O_2_ concentration reached 0.26 ± 0.0032 μmol/g FW, which was 3.25-fold greater than that of the control group. This result indicated that incubation of the X33 antifungal extract induced the oxidative stress in *P. digitatum*.

### Reducing Pyruvate Formation in *P. digitatum* by X33 Antifungal Extract Inducement

Pyruvate is an intermediate involved in the basic metabolism of the whole organism and plays an important role in the metabolic relationships of sugar, fat, and amino acids. To evaluate the effect of *S. lavendulae* strain X33 on pyruvate biosynthesis, the *P. digitatum* hyphae were induced by diverse contents (0, 1.2, 2.4, and 4.8 mg/mL) of the X33 antifungal extract. As a result, the improving content of the X33 antifungal extract caused an obvious decline in pyruvate accumulation in *P. digitatum* (Table 2). When treated with 4.8 mg/mL of the X33 antifungal extract, the pyruvate levels of *P. digitatum* hyphae experienced a 41.41% reduction compared to the control group (2.75 ± 0.18 mg/g FW). The reduction in pyruvate indicated the metabolic disturbance induced by the X33 antifungal extract treatment.

**TABLE 2:** Effect of various concentrations of antifungal extract from *S. lavendulae* strain X33 on pyruvate contents in *P. digitatum.*

**Concentrations of X33 antifungal extract (mg/mL)**	**Pyruvate content (mg/g)**	**Content reduction rate/%**
1.2	2.29 ± 0.13^*d*^	16.68
2.4	1.82 ± 0.08^*c*^	33.71
4.8	1.61 ± 0.06^*b*^	41.41
0 (ck)	2.75 ± 0.18^*a*^	0

*Ck represents the control group treated by sterile waters. Different lowercase letters indicate significant difference (*p* < 0.05).*

## Discussion

Green mold decay is a serious disease in fruit, particularly citrus fruits (Macarisin et al., 2007). Recently, some pathogenic strains have developed resistance against synthetic fungicides, causing concern regarding environmental and food safety. This is an urgent problem that requires the exploration of new environmental and food safety microbial antifungal agents, particularly those produced by Actinomycetes (especially *Streptomyces* spp.). Previously, we screened a *S. lavendulae* strain that inhibited the growth of *P. digitatum* and isolated an active X33 antifungal extract. The present study highlights the protein expression and physiological analysis of *P. digitatum* to investigate the molecular mechanism of the X33 antifungal substance.

### The X33 Antifungal Extract Affects the Mycelial Growth and Morphology of *P. digitatum*

The lethal effect of drugs on causative agent is frequently depending on the degree of drug exposure (time and concentration). The current knowledge suggested that the ZOI of *P. digitatum* increased in a dose-dependent manner, indicating significant inhibition of the growth of *P. digitatum* by the X33 antifungal extract. In addition to the antifungal agents thymol (Perezalfonso et al., 2012), chitosan oligomers (Lu et al., 2014), clove essential oil (Chen et al., 2019a), and trans-cinnamaldehyde (Huang et al., 2019), this study suggests a novel fungicide to control citrus postharvest green mold. Compared to other antifungal agents, the X33 antifungal extract, a poly-L-lysine analog derived from *S. lavendulae*, exhibited excellent antifungal activity. However, microorganisms are extremely adaptable as they require the ability to swiftly phenotypically adapt to a variety of changing environmental factors in order to survive (Burchmore, 2014). If causative agent is not killed by drug exposure, adaptive responses will reduce the drug toxicity to this organism. Uncovering the mechanism of the X33 antifungal extract on the mycelial growth and morphology of *P. digitatum* is beneficial to delay the process of adaptive responses.

The subcellular level analyses showed a clear difference in the hyphae of *P. digitatum* between the X33 antifungal extract treatment and control groups. This may be related to the modulations in membrane permeability regulated by the synthesis of ergosterol, which is a lipid molecule widely listed in the cell membrane and regulates membrane-dependent properties. In addition, reducing the accumulation of ergosterol is frequently lead to the modulations in cell wall. Induced by X33 antifungal extract, ergosterol is unable to maintain cell wall integrity for *P. digitatum*, which caused leakage of cell compounds and, ultimately, hyphae growth restriction or cell death. The changes in mycelial morphology can be connected to the internalization of the X33 antifungal extract into the cell, which interferes with metabolic processes by destroying proteins and enzymes derived in the low viability spores.

### Energy Metabolism in *P. digitatum* Induced by the X33 Antifungal Extract

Proteomics analysis suggested that the X33 antifungal extract induced a cellular response and clearly mechanisms that differed extensively between the treatment and control groups. An appropriate response to these adversities caused by the X33 antifungal extract includes physiological and developmental changes in energy metabolism, the redox system, and cell integrity-related pathways. A considerable number of DEPs related to glycolysis metabolism, such as phosphoglycerate kinase (PGK) (gi| 731318940), aldose 1-epimerase (gi| 859269367), and phosphopyruvate hydratase (gi| 901823486), are upregulated by X33 antifungal extract treatment (Table 1). PGK is the primary ATP-generating enzyme listed in the glycolytic pathway that catalyzes the transfer of phosphate from 1,3-diphosphoglycerate to ADP. Phosphopyruvate hydratase is a widespread enzyme that catalyzes the dehydration of glycerate-2-phosphate to enolpyruvate phosphate. The accelerated glycolysis pathway induced by the X33 antifungal extract might be favored for providing more backbone for other anabolism processes. This is according to a previous study, which reported that β-aminobutyric acid treatment upregulated several proteins involved in the glycolysis pathway, while no significant changes in the ATP synthesis of mango fruit were identified (Li et al., 2019).

Mitochondria is an important organelle involved in aerobic respiration, which generated a multitude of energy, originated from the couple reactions of oxidative phosphorylation and the citric acid cycle, for cellular process and participate in apoptosis process (Shaughnessy et al., 2014). In this work, numerous proteins related to the citric acid cycle and mitochondrial respiratory chain, such as succinate dehydrogenase (SDH) (gi| 859265321) and pyruvate carboxylase (gi| 859268618), were significantly downregulated in *P. digitatum* cells treated with the X33 antifungal extract (Table 1). This phenomenon was consistent with the results of a previous study, which suggested that ethanol extraction of propolis decreased the activities of malate dehydrogenase and SDH, disrupting the tricarboxylic acid cycle in *Penicillium notatum* (Xu et al., 2019).

X33 antifungal extract treatment significantly upregulated the expression of the acetyl-coenzyme A transporter 1 (gi| 859264692), ATP synthase subunit C (gi | 121713168), ATP synthase subunit H (gi| 859270273), nicotinamide adenine dinucleotide (NADH) dehydrogenase 12 kda subunit (gi| 358373893), NADH dehydrogenase (gi| 525588153), NADH-ubiquinone oxidoreductase 20.9 kDa subunit (gi| 952549797), and acyl carrier protein (gi| 995934404) (Table 1). In a previous study, Zhou et al. (Zhou B. et al., 2020) found that *Monascus* spp. can balance the levels of the intracellular redox state and metabolism of materials and energy by activating alternative respiratory pathways associated with NADH dehydrogenase. This resulted in the upregulation of proteins associated with NADH dehydrogenase alternative respiratory pathways, including NADH-ubiquitin dehydrogenase 24 kDa subunits, NADH-quinone oxidoreductase, 40 ubiquitin-protein, and Chromatin structure remodeling complex subunits (Zhou B. et al., 2020). Moreover, lower pyruvate contents were exclusively observed in the X33 antifungal extract-treated samples. Together, these results suggested that the X33 antifungal extract affects the expression of proteins associated with glycolysis, the TCA cycle, and respiratory chain, thereby disturbing the TCA cycle, interrupting energy metabolism, and inducing mitochondrial dysfunction in *P. digitatum*.

### Stress Response Changes in *P. digitatum* Induced by the X33 Antifungal Extract

The dysfunction of mitochondria destroyed electron transport chain that causes the generations of ROS and cellular oxidative stress (OuYang et al., 2018). To protect cells from oxidative stress and maintain normal cellular functions, *P. digitatum* scavenges excess ROS through ROS scavenging enzymes [catalase, peroxidase, and superoxide dismutase (SOD)]. Interestingly, in the present study, SOD [Cu-Zn] (gi| 145237624) and peroxidase (POD) (gi| 859264108) were downregulated, while catalase (CAT) (gi| 584410839) was upregulated in cells treated with the X33 antifungal extract (Table 1). Previous studies have revealed that cellular oxidative stress can induce opposite changes in SOD, POD, and CAT activities (Yao et al., 2020). The modifications in the expression of antioxidant enzyme result in increases in ROS levels and stimulating the defense mechanisms (Jy et al., 2017). It is, thus, possible that the redox system of *P. digitatum* is activated by X33 antifungal extract treatment. Thus, we evaluated the activities of the H_2_O_2_ main compounds associated with ROS. After exposure to the X33 antifungal extract, the impressive improvement in the activities of H_2_O_2_ demonstrates that X33 antifungal extract induced the increases in free radicals, which caused oxidative stress. In *Penicillium expansum* cells, cinnamaldehyde and citral combination treatment stimulates oxidative stress, as demonstrated by the reduction in the activities of CAT and increase in the activities of SOD and H_2_O_2_ (Wang et al., 2018). Xu et al. (2017) reported that tea tree oil treatment improved the content of H_2_O_2_, which is probably lead to oxidative stress and cell membrane changes. Therefore, these results confirmed the speculation that the X33 antifungal extract can target mitochondria and induce oxidative stress in *P. digitatum*.

### Cell Membrane Changes in *P. digitatum* Induced by the X33 Antifungal Extract

Accumulation of ROS is able to affects the normal cellular and membrane functions through oxidizing lipids, nucleic acids, proteins, and carbohydrates (Guo et al., 2019). Phosphatidylcholine and ergosterol are two of the major components of the *P. digitatum* cell membrane. A previous study reported that citral destroys cell membrane integrity, either directly, or indirectly by interfering with cell membrane-related pathways, especially the biosynthesis pathway of fatty acid. In the present study, phosphatidylglycerol-specific phospholipase C (gi| 119482768) and C-4 methylsterol oxidase (gi| 859268431) were upregulated, which are involved in inositol phosphate metabolism and fatty acid metabolism (Table 1). In contrast, the expressions of acetyl-CoA C-acyltransferase (gi| 145258080), 3-β-hydroxysteroid dehydrogenase (gi| 859260572), and acyl-CoA dehydrogenase (gi| 859263266) were downregulated by 1.07- to 1.72-fold. These results imply that the X33 antifungal extract probably affects the integrity of plasma membranes of *P. digitatum* cells through disturbing the biosynthesis of membrane lipid biosynthesis.

To further verify this point, the physiological indexes closely involving in the membrane structure and function were further assayed, such as the extracellular conductivity values, the lipid peroxidation, and the ergosterol accumulation. The enhancement of MDA, a key marker involved in lipid peroxidation, also supported this viewpoint, as its levels significantly increased after the addition of the X33 antifungal extract. The significant increase in the extracellular conductivity values further confirmed the hypothesis that addition of X33 antifungal extract damages membrane integrity by disturbing the biosynthesis of membrane lipid biosynthesis. Based on the results of the above DEPs levels and physiological parameters, membrane structure may be the main target of X33 antifungal extract.

### Cell Wall Change in *P. digitatum* Induced by the X33 Antifungal Extract

The cell wall is the first barrier of filamentous fungi resist environmental pressures and is crucial for maintaining the normal growth of fungal (Gandía et al., 2019). In the current study, chitin synthase (gi| 859266290), endoglucanase (gi| 859262625), 1,3-β-glucanosyltransferase (gi| 859262943), and β-glucosidase (gi| 859260053), which are important cell wall-related proteins, were overexpressed. A previous study reported a similar phenomenon wherein limonene treatment caused a compensatory response to cell wall damage through the overexpression of several genes, which participate in the cell wall integrity signaling pathway.

A significant increase in AKP activity induced by constant X33 antifungal extract exposure time *p* < 0.05; Figure 5B) further confirmed this hypothesis. Cinnamaldehyde was discovered with the ability to inducing the leakage of AKP and damaging the cell wall integrity of *G. citri-aurantii* (OuYang et al., 2019). This finding underlined that the X33 antifungal extract can activate cell wall compensatory mechanisms to overcome X33 antifungal extract toxicity.

## Conclusion

This study presents a proteomics analysis of *P. digitatum* after treatment with the X33 antifungal extract, which performs the novel concept that this extract disturbs the TCA cycle, glycolysis, oxidative phosphorylation, affects the contents of various cellular constitutes, and stimulates cell integrity damage, mitochondrial dysfunction, and oxidative stress of strain *P. digitatum*. These findings not only discovered underlying mechanism for action mode of the X33 antifungal extract on *P. digitatum*, but also provides impressive guidance for the investigation of novel fungicides to reduce the postharvest decay of fruits.

## Data Availability Statement

The raw data supporting the conclusions of this article will be made available by the authors, without undue reservation.

## Author Contributions

S-HL conducted the experiments and wrote the manuscript. PL, EY, and XZ participated in the research. BZ and XW planned, analyzed, and interpreted the data, and wrote the manuscript. All authors contributed to the article and approved the submitted version.

## Conflict of Interest

The authors declare that the research was conducted in the absence of any commercial or financial relationships that could be construed as a potential conflict of interest.

## Footnotes

1 http://www.genome.jp/kegg/
